# Arousal-driven interactions between reward motivation and categorization of emotional facial expressions

**DOI:** 10.3389/fpsyg.2022.985652

**Published:** 2022-11-08

**Authors:** Lakshman N. C. Chakravarthula, Srikanth Padmala

**Affiliations:** Centre for Neuroscience, Indian Institute of Science, Bangalore, KA, India

**Keywords:** emotion, reward, valence, arousal, goal-relevance, facial expressions

## Abstract

Reward motivation and emotion share common dimensions of valence and arousal, but the nature of interactions between the two constructs is relatively unclear. On the one hand, based on the common valence dimension, *valence-compatible* interactions are expected where reward motivation would facilitate the processing of compatible (i.e., positive) emotion and hamper the processing of incompatible (i.e., negative) emotion. On the other hand, one could hypothesize *valence-general* interactions driven by the arousal dimension, where the processing of both positive and negative emotions would be facilitated under reward motivation. Currently, the evidence for valence-compatible vs. valence-general type interactions between reward motivation and goal-relevant emotion is relatively mixed. Moreover, as most of the previous work focused primarily on appetitive motivation, the influence of aversive motivation on goal-relevant emotion is largely unexplored. To address these important gaps, in the present study, we investigated the interactions between motivation and categorization of facial emotional expressions by manipulating the valence dimension of motivation (appetitive and aversive motivation levels) together with that of emotion (positive and negative valence stimuli). Specifically, we conducted two behavioral experiments to separately probe the influence of appetitive and aversive motivation (manipulated *via* an advance cue signaling the prospect of monetary gains in Experiment 1 and losses in Experiment 2, respectively) on the categorization of happy, fearful, and neutral faces. We tested the two competing hypotheses regarding the interactions between appetitive/aversive motivation and emotional face categorization: *Valence-compatible* vs. *Valence-general*. We found evidence consistent with valence-general interactions where both appetitive and aversive motivation facilitated the categorization of happy and fearful faces relative to the neutral ones. Our findings demonstrate that interactions between reward motivation and categorization of emotional faces are driven by the arousal dimension, not by valence.

## Introduction

Reward motivation and emotion share common evaluative dimensions of value/valence and intensity/arousal despite being constructs with distinct mechanisms (Chiew and Braver, 2011; Sander and Nummenmaa, 2021). For instance, Chiew and Braver (2011) succinctly described the relationship between the two constructs as “emotion may … serve to provide an index of value associated with an internal or externally experienced state. While … motivation should be considered a state that produces behavior specifically oriented to carry out a goal that has hedonic value.” However, it was suggested that motivational and emotional manipulations operate in highly similar ways in their impact on perception and cognition (Pessoa, 2009). Following that, proposals have been made for neural correlates of the putative common value/valence dimension of reward motivation and emotion.

A recent functional MRI (fMRI) study, where the authors had included both reward and emotion tasks (in separate blocks) within the same set of participants reported greater activity in a key evaluative brain region for positive compared to negatively valenced stimuli across both domains (Park et al., 2018). Based on this evidence, the authors proposed that activity in the ventro-medial prefrontal cortex may serve as a common neural currency for the coding of the valence dimension of reward and emotion processing. This idea of the common valence dimension can be further extended to hypothesize *valence-compatible* interactions between reward and emotion (Park et al., 2019). For instance, under reward motivation, processing of compatible (i.e., positive) emotion could be facilitated, whereas processing of incompatible (i.e., negative) emotion could be hampered (Park et al., 2019). Alternatively, one could hypothesize *valence-general* interactions between reward and emotion, driven by the arousal dimension. Under this scenario, processing of both positive and negative emotions could be facilitated under reward motivation.

In the scant literature on reward-emotion interactions, one line of work focused on investigating how reward motivation influenced behavior in the presence of task-irrelevant emotional stimuli (Padmala and Pessoa, 2014; Padmala et al., 2017; Walsh et al., 2018, 2019). For instance, Walsh and colleagues employed a letter-search task in an array presented above and below a central negative, positive, or neutral distractor stimulus. The authors reported reward prospect mitigated the interference effect of both positive and negative emotional distractors on task performance to a similar extent (Walsh et al., 2018). These findings indicate that reward motivation countered the deleterious effects of emotional distraction *irrespective* of the valence, thereby providing little support to the hypothesis of valence-compatible interactions between reward motivation and emotion. One plausible reason for not observing valence-compatible interactions might be because arousal rather than the valence dimension of the emotional stimuli is more influential when goal irrelevant (Schimmack and Derryberry, 2005).

However, some researchers have proposed that the valence dimension plays a dominant role when emotional stimuli are goal-relevant (Okon-Singer et al., 2013). So, one could expect that the valence of the emotional stimuli would become more pertinent under such contexts (e.g., in an explicit task of emotion categorization) and thus lead to valence-compatible interactions with reward motivation. Evidence for such valence-compatible interactions using an emotion categorization task was reported previously (Derryberry, 1988). Participants were presented with gain, neutral, and loss reward prospect cues prior to the categorization of emotional words (positive, negative, and neutral categories of valence). It was observed that positive words were categorized faster following gain cues compared to negative words and vice-versa following loss cues. However, a recent study that tested a similar question employing emotional facial expressions failed to observe such a valence-compatible interaction pattern. Wei and Kang (2014) asked participants to categorize angry, neutral, and happy facial expressions preceded by a reward or a no-reward cue. The authors observed that reward motivation interacted with emotion processing such that the categorization of both positive and negative facial expression categories was facilitated relative to the neutral category under the prospect of reward when compared with the absence of such reward prospect. This finding hints at possible *valence-general* interactions between reward motivation and goal-relevant emotion.

Another recent study that brought together the manipulations of reward motivation and goal-relevant emotion was by Park et al. (2019). Unlike in the two studies described above that involved sequential cue-target paradigms, here, the authors explicitly signaled the reward prospect by one of the three emotional facial expression categories (angry, happy, or neutral), and the rest signaled no reward. The particular emotion category that signaled the reward availability varied across three different types of blocks. On each trial, participants were asked to categorize whether a facial stimulus from one of these categories was associated with reward or not, under the premise that it would require integrating reward and emotion information. The authors observed facilitation in response times when categorizing happy faces signaling reward (relative to no reward) prospect, but response times were slowed down when categorizing angry faces signaling reward (relative to no reward) prospect. These findings support the idea of valence-compatible interactions between reward motivation and emotion, where performance benefits were observed when valence-compatible positive faces signaled reward prospect, whereas performance detriments were observed in the case of valence incompatible negative faces.

The mixed nature of findings from the limited set of studies so far precludes any firm conclusions regarding valence-compatible vs. valence-general type interactions between reward motivation and goal-relevant emotion. Moreover, as most of the previous work focused primarily on appetitive motivation (i.e., prospect of monetary gains), the influence of aversive motivation (i.e., prospect of monetary losses) on goal-relevant emotion is largely unexplored (but see Derryberry, 1988). To understand the full breadth of motivation-emotion interactions, it is important to manipulate motivation along the aversive dimension as well (which carries negative value) and study how it impacts positive and negative emotion. Therefore, we sought to investigate the interactions between reward motivation and goal-relevant emotion by manipulating the valence dimension of motivation (appetitive and aversive motivation levels) together with that of emotion (positive and negative valence stimuli). Specifically, we employed the categorization of emotional facial expressions (fearful, happy, and neutral) as a goal-relevant emotion manipulation. Across two experiments, we tested the influence of appetitive and aversive motivation (manipulated *via* an advance cue signaling the prospect of monetary gains and losses, respectively) on categorization of emotional facial expressions. We aimed to test the two competing hypotheses regarding the nature of interactions between appetitive/aversive motivation and emotional face categorization: (i) *Valence-compatible interactions*: categorization of happy faces would be facilitated under appetitive motivation but that of fearful faces would be hindered, and vice versa under the aversive motivation condition; vs. (ii) *Valence-general interactions*: categorization of both happy and fearful faces would be facilitated under both appetitive and aversive motivation conditions.

## Experiment 1

### Methods

#### Demographics

Thirty-six participants from the Indian Institute of Science, Bangalore and other nearby educational institutions volunteered for the study. Data from two participants was excluded because of poor performance (accuracy in one or more of the conditions in the main reward task was at or below chance level). Data from one additional participant was lost due to a technical error. Therefore, data from the remaining 33 participants [17 females; age: 23.5 ± 4.3 years (mean ± SD)] was considered for further analysis. The study was reviewed and approved by the Institutional Human Ethics Committee of Indian Institute of Science, Bangalore. All participants provided written informed consent, had a normal or corrected-to-normal vision, and reported no psychological condition or neurological disorder. Participants were compensated at Rs. 100/h as base pay for their participation.

#### Stimuli

Facial images from 72 identities (36 of each gender) depicting emotional expressions of fearful, neutral, and happy were selected from the following databases: Facial Action Coding Systems (Ekman and Friesen, 1978), The Karolinska Directed Emotional Faces (Lundqvist et al., 1998), NimStim Set of facial expressions (Tottenham et al., 2009), and Radboud Faces Database (Langner et al., 2010). The images were reduced to the visible face area by cropping hair and clothes to mitigate extra-facial features. All images were turned grayscale; the image size and the area occupied by the face were normalized. The average luminance of the images was normalized using the SHINE toolbox for Matlab (Willenbockel et al., 2010). In two pilot studies, self-reported valence and arousal ratings of facial images were collected from two independent groups of 24 participants, each of similar age from the same community. We used a valence-arousal affect grid ratings scale (Russell et al., 1989) with valence dimension on the horizontal axis ranging from 1 (unpleasant) to 9 (pleasant) and arousal dimension on the vertical axis ranging from 1 (low arousal) to 9 (high arousal). The set of 72 identities was considered from two subsets of 36 identities each (of equal gender proportion), rated by each group of 24 participants. The valence and arousal values corresponding to the three emotional expressions of the two sets are reported in Supplementary Table 1; valence and arousal values corresponding to each emotion are comparable between sets. The arousal values for fearful and happy stimuli are also comparable within each set.

#### Procedure

Participants sat in a dark and sound-attenuated room in front of a 19-inch computer screen positioned at a distance of 60 cm. The responses were collected using a standard keyboard. All scripts for stimulus presentation and response collection were written in Matlab version 2019a, using the Psychophysics Toolbox extensions (Brainard and Vision, 1997; Pelli and Vision, 1997; Kleiner et al., 2007). The experimental session was divided into two phases – the Calibration phase and the Main Task phase. The primary purpose of the Calibration phase was to determine the reaction time (RT) threshold per each emotional valence condition separately. This is unlike the design employed in previous studies, where a single, global threshold was used as a reward criterion for all the three valence conditions (Derryberry, 1988; Wei and Kang, 2014). Since mean RT is known to differ across valence conditions (Palermo and Coltheart, 2004; Tottenham et al., 2009), employing a single global threshold might favor some conditions (one with slower RTs) over others (one with faster RTs). Hence, we employed separate RT thresholds for each of the three valence conditions. Moreover, the number of trials per condition used to determine the RT threshold in the Calibration phase was kept similar to the number of trials per condition employed in the Main Task (see below) to derive reliable estimates of reward criteria.

##### Calibration phase

In the Calibration phase, each trial began with an uninformative “##” symbol presented for 1,000 ms, followed by a variable inter-stimulus interval (ISI) between 2 and 6 s. A fixation cross appeared on the screen during the ISI. Then, the face stimulus (5.6° × 8°) with one of the three emotional expressions was presented for 1,000 ms. Participants were provided with a response window of 1,500 ms from the onset of the face stimulus. They were required to press one of the three buttons (numeric keys “1,” “2,” and “3”) corresponding to each emotional expression condition (button mappings were counterbalanced across participants). Finally, a variable inter-trial interval (ITI) between 2 and 6 s with the presentation of a blank screen was employed at the end of the response window, which was followed by the next trial. No feedback about the response was provided to the participant.

The facial stimuli used in the Calibration phase were taken from the first stimulus set of 36 identities, and the other set was used in the Main Task phase (see below; the sets were counterbalanced across participants). Thus, the Calibration phase had 108 trials in total (36 trials per each emotion condition), which were divided into three self-balanced runs of 36 trials each. Separate pseudo-random trial orders were generated per each run such that the same emotional expression and gender identity were repeated not more than twice in a sequence. Once generated, the trial order was fixed same across participants. The ISI and ITI values were sampled from an exponential distribution favoring shorter intervals, and the average ISI value was kept constant across the three emotional expression conditions to control for any potential temporal unpredictability effects (Herry et al., 2007; Koppe et al., 2014).

The Calibration task was preceded by a brief training task of 12 trials, identical to the Calibration phase trials but with additional feedback about accuracy provided at the end of the response window, on each trial. The facial stimuli from a set of four identities (different from the 72-identity-set) were used during training.

For each participant, reward criteria for the subsequent Main Task (see below) were set based on their performance in the Calibration task. The RT threshold value was *separately* calculated for each valence condition by choosing the median RT value of the accurate responses. The prospect of winning a bonus reward in the experiment was revealed only at the end of the Calibration phase so as to avoid such knowledge from impacting the calculation of reward criteria.

##### Main Task phase

**FIGURE 1:**
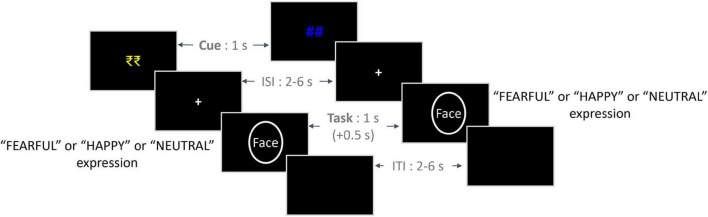
Trial structure of the experimental paradigm. On the left is an exemplar trial from the Reward condition, and on the right is an exemplar trial from the No-reward condition. The initial reward/no-reward cues (1 s) were followed by a jittered ISI (2–6 s), which was followed by a face stimulus (1 s) from either of the three emotion conditions. Participants were given a response window of 1.5 s from the onset of the face stimulus, which included an additional 0.5 s post offset (indicated by +0.5 s). Finally, a jittered ITI (2–6 s) was employed between trials. ISI, inter-stimulus interval; ITI, inter-trial interval.

In the Main Task phase, each trial began with the presentation of a cue indicating the prospect of winning a bonus reward on that trial (reward: “₹₹” in yellow color and no-reward: “##” in blue color; Figure 1) for 1,000 ms. It was followed by a variable ISI period, the presentation of the face stimulus from one of the three emotion conditions, and an ITI – all of them being similar to those employed in the Calibration phase. On trials cued by the “₹₹” symbol (reward condition), participants were provided with a bonus reward of Rs. 1 per trial if they responded accurately and fast enough. For a response to be considered fast, the RT value should be lower than the reward criterion value corresponding to the valence condition (calculated based on the Calibration task). No bonus reward was provided if the RT value exceeded the reward criterion value despite an accurate response. Also, no bonus reward was provided on incorrect responses as well as for no responses within the response window. On trials cued by the “##” symbol (no-reward condition), no bonus reward was provided to the participants. Overall, we employed a 2 *Reward* (reward and no-reward) × 3 *Emotion* (fearful, happy, and neutral) within-subjects design in the Main Task phase.

As mentioned above, disjoint sets of facial stimuli were employed in the Calibration and Main Task phases. All the facial stimuli that appeared in the reward condition were repeated in the no-reward condition. Thus, facial images from 36 identities were used, contributing to 216 trials in total, 36 trials per each of the six experimental conditions. The Main Task phase was divided into 6 self-balanced runs of 36 trials each. Separate pseudo-random trial orders were generated per each run such that the same emotional expression and gender identity were repeated not more than twice in a sequence, and the same reward condition was repeated not more than thrice in a sequence, with the trial order maintained the same across participants. The ISI and ITI values were sampled from an exponential distribution favoring shorter intervals, and the average ISI value was kept constant across the six experimental conditions.

As in the Calibration phase, no feedback about the performance was provided to the participant after every trial. In some previous work, trial-wise reward feedback was shown to influence the emotion recognition in the subsequent trial (Rothermund, 2003), and hence it was not provided. However, at the end of each run, feedback about the reward amount scored in that particular run and the cumulative reward amount won until then was provided. The maximum bonus reward that participants could win in the experiment was Rs. 108. On average, participants earned Rs. 97 (in addition to the base pay of Rs. 150).

The Main Task phase was preceded by a short training phase of 36 trials, identical to the Main Task phase trials but with additional feedback about accuracy and reward information provided at the end of the response window. During reward prospect trials, the following statements were provided as feedback: “Correct and Fast, Won 1” for fast and accurate trials; “Correct but Slow, Won 0” for accurate but slow trials; “Incorrect, Won 0” for incorrect trials and “No Response, Won 0” for trials with no response within the response window. During the trials of no reward condition, accurate trials were indicated by the feedback “Correct, Won 0.” Feedback for incorrect and no response trials was same as that provided during the reward condition. A subset of the facial images used in the Calibration phase were employed during this training task.

#### Data analysis

For the RT analysis of Calibration phase data, error trials (6.71%) and outlier trials (0.81%) with an RT exceeding three standard deviations (SDs) from the condition-specific mean were excluded in each participant. For each participant, mean RT and accuracy rate were determined as a function of *Emotion* (fearful, neutral, and happy), and one-way repeated-measures ANOVAs were conducted in the JASP software (version 0.16.2) (Love et al., 2019). For the RT analysis of Main Task phase data, error trials (7.3%), and outlier trials (0.85%) with an RT exceeding three SDs s from the condition-specific mean were excluded in each participant. For each participant, mean RT and accuracy rate were determined as a function of *Reward* (reward and no-reward) and *Emotion* (fearful, neutral, and happy), and two-way repeated-measures ANOVAs were conducted. In all repeated-measures ANOVA analyses, the Greenhouse–Geisser correction was used to handle deviations from sphericity (Greenhouse and Geisser, 1959). To probe the nature of observed main effects and interaction effects, follow-up pairwise comparisons were conducted by controlling for multiple comparisons using the Holm–Bonferroni method (Holm, 1979). An alpha-level of 0.05 was used for all statistical tests.

### Results

A one-way repeated-measures ANOVA was performed on the RT data from the Calibration phase (Table 1) with *Emotion* (fearful, happy, and neutral) as factor. A main effect of *Emotion* was detected [*F*(1.697,54.309) = 20.412, *p* < 0.001, $\eta_{\text{p}}^{2}=0.389$), with participants being fastest in the happy condition (mean: 819 ms, SD: 105 ms), followed by the neutral condition (mean: 860 ms, SD: 106 ms), and slowest in the fearful condition (mean: 908 ms, SD: 135 ms). The *post hoc* pair-wise comparisons revealed differences between all the three conditions [*fearful* vs. *neutral*: *t*(32) = 3.054, *p* = 0.005, Cohen’s *d* = 0.532; *happy* vs. *neutral*: *t*(32) = −3.814, *p* < 0.001, Cohen’s *d* = −0.664; *fearful* vs. *happy*: *t*(32) = 6.005, *p* < 0.001, Cohen’s *d* = 1.045]. These results support the rationale for employing separate reward criteria per each emotion condition. Similar repeated-measures ANOVA on accuracy data (Table 1) detected a main effect of *Emotion* [*F*(2,64) = 5.277, *p* = 0.008, $\eta_{\text{p}}^{2}=0.142$], with participants being less accurate in the fearful condition (mean: 90.6%, SD: 9.7%) as compared to the neutral (mean: 94.5%, SD: 8.8%), and the happy (mean: 94.8%, SD: 8.15%) conditions, as reflected in the *post hoc* pair-wise comparisons [*fearful* vs. *neutral*: *t*(32) = −2.399, *p* = 0.022, Cohen’s *d* = −0.418; *happy* vs. *neutral*: *t*(32) = 0.269, *p* = 0.790, Cohen’s *d* = 0.047; *fearful* vs. *happy*: *t*(32) = −2.545, *p* = 0.016, Cohen’s *d* = −0.443].

**TABLE 1:** Descriptive RT and accuracy values from Experiment 1.

Calibration phase					
Emotion condition	Reaction time (ms)			Accuracy (percent)	
	Mean	SD		Mean	SD
Fear	907.57	135.46		90.57	9.72
Neutral	859.81	106.32		94.53	8.80
Happy	819.46	105.38		94.78	8.15

**Main Task phase**					

**Reward condition**	**Emotion condition**	**Reaction time (ms)**		**Accuracy (percent)**	
		**Mean**	**SD**	**Mean**	**SD**

No-reward	Fear	823.24	92.97	89.70	11.17
	Neutral	777.01	84.2	93.75	8.36
	Happy	744.78	85.09	93.42	7.97
Reward	Fear	704.24	78.23	91.75	8.38
	Neutral	687.27	77.67	92.85	8.25
	Happy	641.26	82.03	94.87	6.10

**FIGURE 2:**
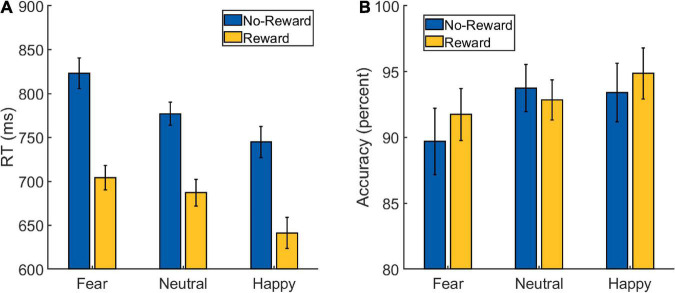
Behavioral data from the Main Task Phase of Experiment 1: **(A)** reaction time (RT) and **(B)** accuracy scores. The error bars represent confidence intervals (±1.96 × within-subject Standard Error) as discussed in O’Brien and Cousineau (2014).

A 2 × 3 repeated-measures ANOVA was performed on the RT data from the Main Task (Figure 2A and Table 1) with *Reward* (reward and no-reward) and *Emotion* (fearful, happy, and neutral) as factors. A main effect of *Reward* was detected [*F*(1,32) = 137.273, *p* < 0.001, $\eta_{\text{p}}^{2}=0.811$], with the participants being faster in the reward (mean: 678 ms, SD: 72 ms) compared to the no-reward condition (mean: 782 ms, SD: 80 ms). A main effect of *Emotion* was also detected [*F*(1.654,52.936) = 30.929, *p* < 0.001, $\eta_{\text{p}}^{2}=0.491$], with participants being fastest in the happy condition (mean: 693 ms, SD: 78 ms), followed by the neutral condition (mean: 732 ms, SD: 76 ms) and slowest in the fearful condition (mean: 764 ms, SD: 79 ms). The *post hoc* pair-wise comparisons revealed differences between all the three conditions [*fearful* vs. *neutral*: *t*(32) = 4.761, *p* < 0.001, Cohen’s *d* = 0.829; *happy* vs. *neutral*: *t*(32) = −3.929, *p* < 0.001, Cohen’s *d* = 0.684; *fearful* vs. *happy*: *t*(32) = 7.062, *p* < 0.001, Cohen’s *d* = 1.229].

Critically, the primary interest to our research question was the interaction between *Reward* and *Emotion* factors, which was also detected [*F*(1.604,51.320) = 4.465, *p* = 0.023, $\eta_{\text{p}}^{2}=0.122$]. To understand the nature of the interaction effect, we first computed the effect of reward motivation on the processing of each of the three emotion types by subtracting reward RT from no-reward RT corresponding to each of them. Then we ran three planned pairwise comparisons that revealed differences in reward effects of fearful (mean: 119 ms, SD: 65 ms) vs. neutral (mean: 90 ms, SD: 56 ms) conditions, whereas the difference of happy (mean: 103 ms, SD: 60 ms) vs. neutral conditions was marginal [*fearful* vs. *neutral*: *t*(32) = 2.483, *p* = 0.018, Cohen’s *d* = 0.432; *happy* vs. *neutral*: *t*(32) = 1.835, *p* = 0.076, Cohen’s *d* = 0.319; *fearful* vs. *happy*: *t*(32) = 1.608, *p* = 0.118, Cohen’s *d* = 0.28].

As we observed a main effect of *Emotion* such that the RT was fastest during the happy condition, it is conceivable that the reward effects during the happy (vs. neutral) condition were weaker because of faster RTs as they would leave less room for facilitation. Therefore, we additionally calculated a ratio-based index of facilitation (which takes into account the overall RT differences between conditions) separately for happy (RT _No–Reward, Happy_/RT _Reward, Happy_), fearful (RT _No–Reward, Fearful_/RT _Reward, Fearful_) and neutral (RT _No–Reward, Neutral_/RT _Reward, Neutral_) conditions in each participant. A comparison of these ratio-based indices between conditions *via* paired *t*-test revealed significant difference between fearful and neutral conditions [*t*(32) = 2.085, *p* = 0.045, Cohen’s *d* = 0.363] and between happy and neutral conditions [*t*(32) = 2.49, *p* = 0.018, Cohen’s *d* = 0.433], but no difference was detected between fearful and happy conditions [*t*(32) = 0.358, *p* = 0.723, Cohen’s *d* = 0.062]. These results further support the interpretation of increased facilitation during the happy and fearful conditions. Overall, the categorization of fearful and happy faces was facilitated by reward motivation compared to neutral faces.

A similar 2 × 3 repeated-measures ANOVA was performed on the accuracy data (Figure 2B and Table 1) with *Reward* (reward and no-reward) and *Emotion* (fearful, happy, and neutral) as factors. A main effect of *Emotion* was detected [*F*(1.710,54.726) = 4.210, *p* = 0.019, $\eta_{\text{p}}^{2}=0.116$], with *post hoc* pair-wise comparisons revealing difference only between *fearful* (mean: 90.75%, SD: 9.13%) and *happy* (mean: 94.15%, SD: 6%) conditions [*fearful* vs. *happy*: *t*(32) = −2.785, *p* = 0.021, Cohen’s *d* = 0.485; *fearful* vs. *neutral*: *t*(32) = −2.099, *p* = 0.08, Cohen’s *d* = −0.365; *happy* vs. *neutral*: *t*(32) = 0.686, *p* = 0.495, Cohen’s *d* = 0.119]. The main effect of *Reward* [*F*(1,32) = 0.907, *p* = 0.348, $\eta_{\text{p}}^{2}=0.028$] and the *Reward* × *Emotion* interaction were not detected [*F*(1.677,53.667) = 2.361, *p* = 0.112, $\eta_{\text{p}}^{2}=0.069$].

### Discussion

In the RT data, we observed that the reward motivation facilitated the categorization of all the three emotional categories. However, the extent of facilitation was greater for salient stimuli (fearful and happy facial expressions) compared to the neutral one. This result suggests that appetitive motivation facilitated the categorization of emotional facial expressions in a valence-general manner. Nevertheless, as discussed earlier, a comprehensive picture of motivation-emotion interactions can be obtained by further studying the influence of aversive motivation on goal-relevant emotion.

## Experiment 2

We conducted Experiment 2 by replacing appetitive motivation with aversive motivation to investigate its interaction with emotional face categorization. We ensured that the structure of the experiment remained as close as possible to that of Experiment 1. To iterate the hypotheses in the light of the results of Experiment 1, we expected that the valence-general interaction hypothesis would be strengthened if aversive motivation also led to greater facilitation in the categorization of happy and fearful faces compared to the neutral ones.

### Methods

A new group of 36 participants was recruited for this experiment. Data from one participant was excluded because of poor performance (accuracy in one or more of the conditions in the main task was at or below chance level). Therefore, data from the remaining thirty-five participants [9 females; age: 23 ± 4.3 years (mean ± SD)] was considered for further analysis. All the methods including the stimuli, trial structure, and procedure (including the Calibration and the Main Task phases) were kept the same except for the following changes. Prior to the start of the Main Task phase, participants were informed that Rs. 108 had been deposited in their account as bonus money that they could retrieve at the end of the experiment. They were then instructed that the trials that began with a rupee symbol as a cue had a prospect of monetary loss of Rs. 1 per trial (from the deposited bonus money) if they were not accurate and fast enough. Trials that began with “##” symbol had no prospect of monetary loss. The sum of money lost in each run, along with the cumulative loss amount, was displayed to the participant after every run. On average, participants retained Rs. 88 of bonus money (in addition to the base pay of Rs. 150).

#### Data analysis

For the RT analysis of Calibration phase data, error trials (5.61%) and outlier trials (0.87%) with an RT exceeding three SDs s from the condition-specific mean were excluded in each participant. For the RT analysis of Main Task phase data, error trials (8.32%) and outlier trials (0.65%) with an RT exceeding three SDs from the condition-specific mean were excluded in each participant. The rest of the analysis procedures were same as in Experiment 1 where a one-way repeated-measures ANOVA was conducted on the Calibration phase data, and a two-way repeated-measures ANOVA was conducted on the Main Task phase data.

### Results

One-way repeated-measures ANOVA was performed on the RT data from the Calibration phase (Table 2) with *Emotion* (fearful, happy, and neutral) as factor yielded a main effect of *Emotion* [*F*(1.661,56.471) = 25.087, *p* < 0.001, $\eta_{\text{p}}^{2}=0.425$], with participants being fastest in the happy condition (mean: 742 ms, SD: 80 ms), followed by the neutral condition (mean: 798 ms, SD: 94 ms) and slowest in the fearful condition (mean: 824 ms, 66 ms). The *post hoc* pair-wise comparisons revealed differences between all the three conditions [*fearful* vs. *neutral*: *t*(34) = 2.407, *p* = 0.022, Cohen’s *d* = 0.407; *happy* vs. *neutral*: *t*(34) = −3.920, *p* < 0.001, Cohen’s *d* = −0.663; *fearful* vs. *happy*: *t*(34) = 8.295, *p* < 0.001, Cohen’s *d* = 1.402]. Similar repeated-measures ANOVA on accuracy data (Table 2) yielded only a marginal main effect of *Emotion* [*F*(1.604,54.550) = 2.810, *p* = 0.08, $\eta_{\text{p}}^{2}=0.076$].

**TABLE 2:** Descriptive RT and accuracy values from Experiment 2.

Calibration phase					
Emotion condition	Reaction time (ms)			Accuracy (percent)	
	Mean	SD		Mean	SD
Fear	823.72	65.57		93.17	9.49
Neutral	797.57	93.7		93.89	5.30
Happy	742.37	79.63		96.11	4.05

**Main Task phase**					

**Loss condition**	**Emotion condition**	**Reaction time (ms)**		**Accuracy (percent)**	
		**Mean**	**SD**	**Mean**	**SD**

No-loss	Fear	763.35	73.35	86.67	13.03
	Neutral	721.0	71.84	91.75	7.84
	Happy	701.4	80.96	91.51	9.06
Loss	Fear	678.21	63.09	91.83	7.50
	Neutral	660.35	63.8	94.21	6.11
	Happy	618.51	80.22	94.13	6.52

**FIGURE 3:**
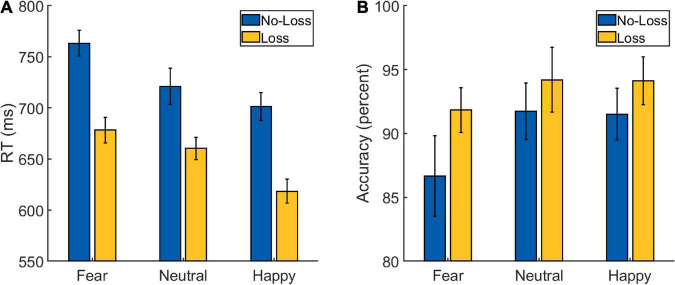
Behavioral data from the Main Task Phase of Experiment 2: **(A)** reaction time (RT) and **(B)** accuracy scores. The error bars represent confidence intervals (±1.96 × within-subject Standard Error) as discussed in O’Brien and Cousineau (2014).

A 2 × 3 repeated-measures ANOVA was performed on the RT data from the Main Task (Figure 3A and Table 2) with *Loss* (loss and no-loss) and *Emotion* (fearful, happy, and neutral) as factors. A main effect of *Loss* was detected [*F*(1,34) = 166.597, *p* < 0.001, $\eta_{\text{p}}^{2}=0.831$], with the participants being faster in the loss (mean: 652 ms, SD: 64 ms) compared to the no-loss condition (mean: 729 ms, SD: 66 ms). A main effect of *Emotion* was also detected [*F*(2,68) = 25.763, *p* < 0.001, $\eta_{\text{p}}^{2}=0.431$], with participants being fastest in the happy condition (mean: 660 ms, SD: 78 ms), followed by the neutral condition (mean: 691 ms, SD: 64 ms), and slowest in the fearful condition (mean: 721 ms, SD: 64 ms). The *post hoc* pair-wise comparisons revealed differences between all the three conditions [*fearful* vs. *neutral*: *t*(34) = 3.512, *p* = 0.001, Cohen’s *d* = 0.594; *happy* vs. *neutral*: *t*(34) = −3.239, *p* = 0.003, Cohen’s *d* = −0.548; *fearful* vs. *happy*: *t*(34) = 8.436, *p* < 0.001, Cohen’s *d* = 1.426].

More importantly, the interaction between *Loss* and *Emotion* factors was also detected [*F*(2,68) = 5.047, *p* = 0.009, $\eta_{\text{p}}^{2}=0.129$] which is of primary interest. To further examine the nature of this interaction effect, we first computed the effect of aversive motivation (loss effect) by subtracting loss RT from no-loss RT separately for each of the three emotion conditions. Then, we ran three planned pairwise comparisons that yielded differences in loss effects of fearful (mean: 85 ms, SD: 48 ms) vs. neutral (mean: 61 ms, SD: 48 ms) conditions, and of happy (mean: 83 ms, SD: 40 ms) vs. neutral ones [*fearful* vs. *neutral*: *t*(34) = 2.948, *p* = 0.006, Cohen’s *d* = 0.498; *happy* vs. *neutral*: *t*(34) = 2.627, *p* = 0.013, Cohen’s *d* = 0.444; *fearful* vs. *happy*: *t*(34) = 0.255, *p* = 0.8, Cohen’s *d* = 0.043].

As in Experiment 1, we additionally calculated a ratio-based index of facilitation (which takes into account the overall RT differences between conditions) separately for happy (RT _No–Loss, Happy_/RT _Loss, Happy_), fearful (RT _No–Loss, Fearful_/RT _Loss, Fearful_), and neutral (RT _No–Loss, Neutral_/RT _Loss, Neutral_) conditions in each participant. A comparison of these ratio-based indices between conditions *via* paired *t*-test revealed significant difference between fearful and neutral conditions [*t*(34) = 2.752, *p* = 0.009, Cohen’s *d* = 0.465] and between happy and neutral conditions [*t*(34) = 3.290, *p* = 0.002, Cohen’s *d* = 0.556], but no difference was detected between fearful and happy conditions [*t*(34) = −0.738, *p* = 0.465, Cohen’s *d* = −0.125]. Overall, these results indicate that the categorization of fearful and happy faces was facilitated by loss expectancy compared to that of neutral faces.

A 2 *Loss* (loss and no-loss) × 3 *Emotion* (fearful, happy, and neutral) repeated-measures ANOVA performed on the accuracy data (Figure 3B and Table 2) resulted in a main effect of *Loss* [*F*(1,34) = 10.221, *p* = 0.003, $\eta_{\text{p}}^{2}=0.231$], with the participants being more accurate in the loss (mean: 93.5%, SD: 4.73%) compared to the no-loss condition (mean: 90%, SD: 8.14%). A main effect of *Emotion* was also detected [*F*(2,68) = 4.671, *p* = 0.013, $\eta_{\text{p}}^{2}=0.121$] with greater accuracy in the Happy (mean: 92.8%, SD: 7.05%) and Neutral (mean: 93%, SD: 5.87%) conditions compared to the Fearful (mean: 89.2%, SD: 9.24%) one [*fearful* vs. *neutral*: *t*(34) = −2.405, *p* = 0.022, Cohen’s *d* = −0.407; *happy* vs. *neutral*: *t*(34) = −0.115, *p* = 0.909, Cohen’s *d* = −0.019; *fearful* vs. *happy*: *t*(34) = −3.027, *p* = 0.005, Cohen’s *d* = −0.512]. However, a significant interaction effect was not detected [*F*(2,68) = 1.598, *p* = 0.210, $\eta_{\text{p}}^{2}=0.045$].

### Discussion

The RT interaction results from the Main Task phase were similar to those observed in Experiment 1, with aversive motivation leading to greater facilitation in the categorization of both fearful and happy faces relative to facilitation in the categorization of neutral faces. This complementary evidence further supports the hypothesis of valence-general interactions between reward motivation (specifically aversive motivation, in this experiment) and emotional face categorization.

#### Analysis of reaction times across Experiments 1 and 2

Finally, we statistically compared the RT data across two experiments by running a three-way mixed ANOVA with *Motivation* and *Emotion* as within-subject factors and *Experiment* as the between-subjects factor. We observed significant main effects of Motivation [*F*(1,66) = 291.942, *p* < 0.001, $\eta_{\text{p}}^{2}=0.222$], Emotion [*F*(2,132) = 56.744, *p* < 0.001, $\eta_{\text{p}}^{2}=0.079$], and Experiment [*F*(1, 66) = 5.763, *p* < 0.019, $\eta_{\text{p}}^{2}=0.042$] factors. In terms of two-way interactions, we observed significant Motivation × Emotion [*F*(2,132) = 8.956, *p* < 0.001, $\eta_{\text{p}}^{2}=0.003$] and Motivation × Experiment [*F*(1,66) = 6.968, *p* < 0.010, $\eta_{\text{p}}^{2}=0.005$] interactions, but Emotion × Experiment interaction [*F*(2,132) = 0.373, *p* = 0.689, $\eta_{\text{p}}^{2}=0.0005$] was absent. Of primary interest, the three-way interaction between Motivation, Emotion, and Experiment was not detected [*F*(2,132) = 0.537, *p* = 0.586, $\eta_{\text{p}}^{2}=0.0002$]. The presence of a significant Motivation × Emotion interaction along with the non-significant three-way interaction suggests that both appetitive (in Experiment 1) and aversive (in Experiment 2) motivation facilitated the categorization of fearful and happy faces relative to the neutral ones in a comparable manner.

## General discussion

Our current behavioral investigation aimed at disambiguating between possible valence-compatible and valence-general interactions between motivation and goal-relevant emotion. We observed support for valence-general interactions across two experiments probing for the impact of appetitive and aversive motivation respectively, on categorization of emotional facial expressions. Specifically, in the RT data, we observed that both the manipulations of appetitive and aversive motivation (representing positive and negative value associated with the future prospect of monetary incentives) facilitated the categorization of both happy and fearful faces (representing positive and negative valence associated with the emotional stimuli) relative to that of neutral faces.

In the first experiment, by manipulating appetitive motivation *via* an advance cue that signaled the prospect of performance-based monetary gains, we observed greater performance benefits on happy and fearful faces compared to the neutral ones. These findings are consistent with the ones previously reported by Wei and Kang (2014), where the authors observed greater facilitation effects of reward motivation on categorization of happy and angry faces relative to the neutral faces. To further confirm the pattern of valence-general interactions observed in the first experiment, we conducted a second experiment by replacing appetitive with aversive motivation. Even when participants were motivated to perform fast and accurate to avoid monetary losses, greater RT facilitation was observed on happy and fearful faces relative to the neutral ones. The consistency of the RT interaction pattern across both experiments provide strong support for the valence-general type interactions between appetitive/aversive motivation and categorization of emotional facial expressions. These findings indicate that the observed interactions were primarily driven by the arousal dimension of motivation and emotion. In line with some recent studies that extended the framework of arousal biased competition model (Mather and Sutherland, 2011) to reward-emotion contexts (Kim and Anderson, 2020; Wang et al., 2021), appetitive and aversive motivation-driven arousal might have led to faster categorization of salient emotional stimuli over neutral ones.

Our results are not consistent with the competing hypothesis of valence-compatible interactions between motivation and emotion reported previously (Derryberry, 1988). Although the reasons for the difference in the nature of the interactions are not apparent, a few observations can be made. Firstly, Derryberry (1988) manipulated appetitive and aversive motivation within the same experiment, which might have increased the relative contrast along the value dimension between the motivation conditions. The appetitive motivation condition (prospect of monetary gains) could have been perceived to be inducing more positive value when occurring alongside an aversive motivation condition (prospect of monetary losses) and vice-versa. That is unlike the current study, where appetitive and aversive motivation were manipulated in separate experiments alongside the corresponding no-gain/no-loss control conditions. Secondly, unlike in the present study, Derryberry (1988) provided distinct feedback of success or failure after every trial corresponding to each motivation condition. In such a scenario, processing feedback signals that elicit positive or negative affect (Berridge and Robinson, 2003) would influence the processing of both the motivational cue and emotional stimulus in the subsequent trial (Derryberry, 1993; Rothermund, 2003). Hence, the findings reported in Derryberry (1988) might be specific to contexts where outcome states were also manipulated in addition to motivational and emotional manipulations. Finally, a close inspection of the evidence for valence-compatible interaction between motivation and emotion reported in Derryberry (1988) revealed that only a subset of conditions (ignoring the control/neutral conditions of both the motivation and emotion manipulations) were considered for the statistical interaction. Thus, restricting the analysis to the conditions at the extreme ends of the value/valence manipulations might have favored the detection of valence-compatible interactions. Whereas in the current study, the consideration of neutral conditions and their comparison against positive/negative conditions of both the manipulations served as the key to the detection of valence-general interactions. In any case, how these differences in the design and analysis choices might lead to divergent interaction patterns needs to be further investigated using close replications.

One recent fMRI study that combined the manipulations of reward motivation and goal-relevant emotion reported behavioral signatures of valence-compatible interactions (Park et al., 2019). In this study, the authors manipulated reward motivation in a *reactive* fashion where one of the three emotional categories was used to explicitly signal reward prospect. This kind of reactive reward manipulation where a feature of the target stimulus (an emotional category in this particular study) was used to signal reward availability, is different from the proactive reward manipulation that we employed in the present study where a cue in advance of the task phase indicated reward opportunity. Another critical difference between the design employed in Park et al. (2019) and the current study is the nature of the task. In Park et al. (2019), participants were asked to respond whether or not the emotional face presented during each trial was associated with reward or not. A response to such a task includes processing the emotional expression, making it goal-relevant. However, it also includes subsequent processing of the association between the emotion and reward categories prior to planning an appropriate motor response. Thus, the response time reflects a relatively complex process compared to a simple emotion categorization task employed in the current study. It is possible that the reported valence-compatible interactions between reward and emotion processing in Park et al. (2019) may emerge from the later association stage as opposed to emotional categorization *per se*. Such a possibility also highlights the difference in the design element of incorporating a pairing between emotion and reward categories, which is absent by nature in a *proactive* reward paradigm like the one employed in the current study. Further research is required to probe the necessity of employing explicit associations between emotion and reward categories for valence-compatible interactions to manifest. More generally, a careful comparison between the two modes (proactive and reactive) in which reward motivation can impact goal-relevant emotion needs to be further investigated.

Another set of studies that specifically probed the impact of reward motivation on task-irrelevant emotional stimuli (Padmala and Pessoa, 2014; Padmala et al., 2017; Walsh et al., 2018) reported that reward motivation countered the adverse impact of emotional distraction on task performance. In particular, Walsh et al. (2018) reported that reward motivation mitigated the emotional distraction resulting from both the positive and negative stimuli, reflecting the valence-general nature of interactions between reward motivation and goal-irrelevant emotion processing. Similar findings were also recently reported in the context of a memory task that involved reward motivation and task-irrelevant emotional manipulations during the encoding phase (Wang et al., 2021). In comparison, the current and earlier studies that employed goal-relevant emotional manipulations reported *facilitatory* as opposed to *competitive* interactions reported when emotional stimuli were goal-irrelevant. Nevertheless, independent of whether emotional processing was goal-relevant or goal-irrelevant, the nature of interactions with reward motivation remained valence-general, guided by arousal rather than the valence of the emotional stimuli. The inference made here about the valence-general nature of interactions might be specific to perceptual tasks, as some studies have reported valence-compatible interactions during decision-making (Talmi et al., 2009; Park et al., 2011).

Regarding plausible brain mechanisms, functional interactions between reward and emotion processing regions could underlie the observed valence-general interactions between reward motivation and goal-relevant emotion. Separate lines of neuroimaging research have frequently implicated sub-cortical regions such as the ventral striatum and amygdala in reward motivation and emotion processing, respectively (Knutson and Cooper, 2005; Sergerie et al., 2008). Specifically, the ventral striatum is reported to be involved during the processing of both gain and loss prospect cues reflecting motivational salience (Carter et al., 2009; Oldham et al., 2018), and the amygdala is reported to be involved in the processing of salient emotional faces independent of their valence (Breiter et al., 1996; Sabatinelli et al., 2011). Moreover, these two sub-cortical regions are anatomically connected, providing a pathway for direct communication (Haber and Knutson, 2010). Based on these observations, we hypothesize that interactions between the ventral striatum and amygdala might have played a key role in leading to the observed behavioral interaction pattern. Additionally, top-down attentional regions might have mediated the influence of reward motivation stemming from the ventral striatum on emotional processing in the amygdala. This is consistent with the idea that reward expectancy-driven top-down attention might have biased the processing of salient emotional faces over the neutral ones (Wei and Kang, 2014). Future neuroimaging work could investigate these and other potential brain mechanisms underlying the observed behavioral interaction pattern.

## Conclusion

In conclusion, we observed renewed support for valence-general interactions between motivation and goal-relevant emotion, which can further bridge the disparate literature on reward and emotion to understand the mechanisms of their interaction (Padmala et al., 2019). Such pieces of evidence may further help our understanding of affective disorders with symptoms reflecting abnormal reward and emotional processing.

## Data availability statement

The raw data supporting the conclusions of this article will be made available by the authors, without undue reservation.

## Ethics statement

The studies involving human participants were reviewed and approved by the Institutional Human Ethics Committee of Indian Institute of Science, Bangalore. The patients/participants provided their written informed consent to participate in this study.

## Author contributions

LC and SP designed the research, interpreted the results, and wrote the manuscript. LC performed the data collection and analysis. Both authors contributed to the article and approved the submitted version.

## References

[B1] Berridge K. C. Robinson T. E. (2003). Parsing reward. *Trends Neurosci.* 26 507–513. 10.1016/S0166-2236(03)00233-9 12948663

[B2] Brainard D. H. Vision S. (1997). The psychophysics toolbox. *Spat. Vis.* 10 433–436. 10.1163/156856897X00357 9176952

[B3] Breiter H. C. Etcoff N. L. Whalen P. J. Kennedy W. A. Rauch S. L. Buckner R. L. (1996). Response and habituation of the human amygdala during visual processing of facial expression. *Neuron* 17 875–887. 10.1016/S0896-6273(00)80219-6 8938120

[B4] Carter R. M. MacInnes J. J. Huettel S. A. Adcock R. A. (2009). Activation in the VTA and nucleus accumbens increases in anticipation of both gains and losses. *Front. Behav. Neurosci.* 3:21. 10.3389/neuro.08.021.2009 19753142 PMC2742668

[B5] Chiew K. S. Braver T. S. (2011). Positive affect versus reward: emotional and motivational influences on cognitive control. *Front. Psychol.* 2:279. 10.3389/fpsyg.2011.00279 22022318 PMC3196882

[B6] Derryberry D. (1988). Emotional influences on evaluative judgments: Roles of arousal, attention, and spreading activation. *Motiv. Emot.* 12 23–55. 10.1007/BF00992471

[B7] Derryberry D. (1993). Attentional consequences of outcome-related motivational states: Congruent, incongruent, and focusing effects. *Motiv. Emot.* 17 65–89. 10.1007/BF00995186

[B8] Ekman P. Friesen W. V. (1978). *Facial Action Coding System: A Technique for the Measurement of Facial Movement.* Palo Alto: Consulting Psychologists Press. 10.1037/t27734-000

[B9] Greenhouse S. W. Geisser S. (1959). On methods in the analysis of profile data. *Psychometrika* 24 95–112. 10.1007/BF02289823

[B10] Haber S. N. Knutson B. (2010). The reward circuit: linking primate anatomy and human imaging. *Neuropsychopharmacology* 35 4–26. 10.1038/npp.2009.129 19812543 PMC3055449

[B11] Herry C. Bach D. R. Esposito F. Di Salle F. Perrig W. J. Scheffler K. (2007). Processing of temporal unpredictability in human and animal amygdala. *J. Neurosci.* 27 5958–5966. 10.1523/JNEUROSCI.5218-06.2007 17537966 PMC6672268

[B12] Holm S. (1979). A simple sequentially rejective multiple test procedure. *Scand. J. Stat.* 6 65–70.

[B13] Kim A. J. Anderson B. A. (2020). Arousal-biased competition explains reduced distraction by reward cues under threat. *eNeuro* 7 ENEURO.99–ENEURO.20. 10.1523/ENEURO.0099-20.2020 32601095 PMC7340842

[B14] Kleiner M. Brainard D. Pelli D. (2007). What’s new in Psychtoolbox-3? *Perception* 36 1–16.

[B15] Knutson B. Cooper J. C. (2005). Functional magnetic resonance imaging of reward prediction. *Curr. Opin. Neurol.* 18 411–417. 10.1097/01.wco.0000173463.24758.f6 16003117

[B16] Koppe G. Gruppe H. Sammer G. Gallhofer B. Kirsch P. Lis S. (2014). Temporal unpredictability of a stimulus sequence affects brain activation differently depending on cognitive task demands. *Neuroimage* 101 236–244. 10.1016/j.neuroimage.2014.07.008 25019681

[B17] Langner O. Dotsch R. Bijlstra G. Wigboldus D. H. Hawk S. T. Van Knippenberg A. (2010). Presentation and validation of the Radboud Faces Database. *Cogn. Emot.* 24 1377–1388. 10.1080/02699930903485076 29927004

[B18] Love J. Selker R. Marsman M. Jamil T. Dropmann D. Verhagen J. (2019). JASP: Graphical statistical software for common statistical designs. *J. Stat. Softw.* 88 1–17. 10.18637/jss.v088.i02

[B19] Lundqvist D. Flykt A. Öhman A. (1998). *The Karolinska Directed Emotional Faces – KDEF, CD ROM From Department of Clinical Neuroscience, Psychology Section, Karolinska Institutet*, ISBN 91-630-7164-9.

[B20] Mather M. Sutherland M. R. (2011). Arousal-biased competition in perception and memory. *Perspective. Psychol. Sci.* 6 114–133. 10.1177/1745691611400234 21660127 PMC3110019

[B21] O’Brien F. Cousineau D. (2014). Representing Error bars in within-subject designs in typical software packages. *Quant. Methods Psychol.* 10, 56–67. 10.20982/tqmp.10.1.p056

[B22] Okon-Singer H. Lichtenstein-Vidne L. Cohen N. (2013). Dynamic modulation of emotional processing. *Biol. Psychol.* 92 480–491. 10.1016/j.biopsycho.2012.05.010 22676964

[B23] Oldham S. Murawski C. Fornito A. Youssef G. Yücel M. Lorenzetti V. (2018). The anticipation and outcome phases of reward and loss processing: A neuroimaging meta-analysis of the monetary incentive delay task. *Hum. Brain Map.* 39 3398–3418. 10.1002/hbm.24184 29696725 PMC6055646

[B24] Padmala S. Pessoa L. (2014). Motivation versus aversive processing during perception. *Emotion* 14:450. 10.1037/a0036112 24708503 PMC4110899

[B25] Padmala S. Sambuco N. Pessoa L. (2019). Interactions between reward motivation and emotional processing. *Prog. Brain Res.* 247 1–21. 10.1016/bs.pbr.2019.03.023 31196430

[B26] Padmala S. Sirbu M. Pessoa L. (2017). Potential reward reduces the adverse impact of negative distractor stimuli. *Soc. Cogn. Affect. Neurosci.* 12 1402–1413. 10.1093/scan/nsx067 28505380 PMC5629819

[B27] Palermo R. Coltheart M. (2004). Photographs of facial expression: Accuracy, response times, and ratings of intensity. *Behav. Res. Methods* 36 634–638. 10.3758/BF03206544 15641409

[B28] Park H. R. Kostandyan M. Boehler C. N. Krebs R. M. (2018). Smiling faces and cash bonuses: exploring common affective coding across positive and negative emotional and motivational stimuli using fMRI. *Cogn. Affect. Behav. Neurosci.* 18 550–563. 10.3758/s13415-018-0587-3 29644568

[B29] Park H. R. Kostandyan M. Boehler C. N. Krebs R. M. (2019). Winning smiles: Signalling reward by overlapping and non-overlapping emotional valence differentially affects performance and neural activity. *Neuropsychologia* 122 28–37. 10.1016/j.neuropsychologia.2018.11.018 30521814

[B30] Park S. Q. Kahnt T. Rieskamp J. Heekeren H. R. (2011). Neurobiology of value integration: when value impacts valuation. *J. Neurosci. Res.* 31 9307–9314. 10.1523/JNEUROSCI.4973-10.2011 21697380 PMC6623498

[B31] Pelli D. G. Vision S. (1997). The VideoToolbox software for visual psychophysics: Transforming numbers into movies. *Spat. Vis.* 10 437–442. 10.1163/156856897X00366 9176953

[B32] Pessoa L. (2009). How do emotion and motivation direct executive control? *Trends Cogn. Sci.* 13 160–166. 10.1016/j.tics.2009.01.006 19285913 PMC2773442

[B33] Rothermund K. (2003). Motivation and attention: Incongruent effects of feedback on the processing of valence. *Emotion* 3:223. 10.1037/1528-3542.3.3.223 14498793

[B34] Russell J. A. Weiss A. Mendelsohn G. A. (1989). Affect grid: a single-item scale of pleasure and arousal. *J. Pers. Soc. Psychol.* 57:493. 10.1037/0022-3514.57.3.493

[B35] Sabatinelli D. Fortune E. E. Li Q. Siddiqui A. Krafft C. Oliver W. T. (2011). Emotional perception: meta-analyses of face and natural scene processing. *Neuroimage* 54 2524–2533. 10.1016/j.neuroimage.2010.10.011 20951215

[B36] Sander D. Nummenmaa L. (2021). Reward and emotion: an affective neuroscience approach. *Curr. Opin. Behav. Sci.* 39 161–167. 10.1016/j.cobeha.2021.03.016

[B37] Schimmack U. Derryberry D. E. (2005). Attentional interference effects of emotional pictures: threat, negativity, or arousal? *Emotion* 5:55. 10.1037/1528-3542.5.1.55 15755219

[B38] Sergerie K. Chochol C. Armony J. L. (2008). The role of the amygdala in emotional processing: a quantitative meta-analysis of functional neuroimaging studies. *Neurosci. Biobehav. Rev.* 32 811–830. 10.1016/j.neubiorev.2007.12.002 18316124

[B39] Talmi D. Dayan P. Kiebel S. J. Frith C. D. Dolan R. J. (2009). How humans integrate the prospects of pain and reward during choice. *J. Neurosci.* 29 14617–14626. 10.1523/JNEUROSCI.2026-09.2009 19923294 PMC2948535

[B40] Tottenham N. Tanaka J. W. Leon A. C. McCarry T. Nurse M. Hare T. A. (2009). The NimStim set of facial expressions: judgments from untrained research participants. *Psychiatry Res.* 168 242–249. 10.1016/j.psychres.2008.05.006 19564050 PMC3474329

[B41] Walsh A. T. Carmel D. Grimshaw G. M. (2019). Reward elicits cognitive control over emotional distraction: Evidence from pupillometry. *Cogn. Affect. Behav. Neurosci.* 19 537–554. 10.3758/s13415-018-00669-w 30488225

[B42] Walsh A. T. Carmel D. Harper D. Grimshaw G. M. (2018). Motivation enhances control of positive and negative emotional distractions. *Psychon. Bull. Rev.* 25 1556–1562. 10.3758/s13423-017-1414-5 29299776

[B43] Wang H. Li Y. Chen J. Liu X. Zhang Q. Chen M. (2021). The interaction between reward and the task-irrelevant emotional context in memory. *Memory* 29 129–140. 10.1080/09658211.2020.1860229 33320037

[B44] Wei P. Kang G. (2014). Task relevance regulates the interaction between reward expectation and emotion. *Exp. Brain Res.* 232 1783–1791. 10.1007/s00221-014-3870-8 24553754

[B45] Willenbockel V. Sadr J. Fiset D. Horne G. O. Gosselin F. Tanaka J. W. (2010). Controlling low-level image properties: the SHINE toolbox. *Behav. Res. Methods* 42 671–684. 10.3758/BRM.42.3.671 20805589

