# Supplementary material for: Arousal-driven interactions between reward motivation and categorization of emotional facial expressions
Source: Front Psychol. 2022 Nov 8;13:985652. doi: 10.3389/fpsyg.2022.985652 (PMC9681152; doi:10.3389/fpsyg.2022.985652)
Supplement: Supplementary file 1 [file Table_1.DOCX]

**“Arousal-driven interactions between reward motivation and categorization of emotional facial expressions”, by Lakshman N.C. Chakravarthula and Srikanth Padmala**

**Supplementary Material**

**Table S1**: Mean self-reported Valence and Arousal ratings (with standard deviation in parentheses) of the two sets of 36 facial stimuli each that were employed in this study

| Emotional Expression | Set 1 | | Set2 | |
| --- | --- | --- | --- | --- |
|  | Valence | Arousal | Valence | Arousal |
| Fearful | 2.89 (0.32) | 6.07 (0.52) | 2.47 (0.35) | 6.79 (0.66) |
| Neutral | 4.81(0.31) | 4.09 (0.35) | 4.7 (0.45) | 3.94 (0.3) |
| Happy | 7.35 (0.42) | 6.31 (0.49) | 7.39 (0.43) | 6.23 (0.64) |
